# Carriership of two copies of *C9orf72* hexanucleotide repeat intermediate-length alleles is a risk factor for ALS in the Finnish population

**DOI:** 10.1186/s40478-020-01059-5

**Published:** 2020-11-09

**Authors:** Karri Kaivola, Samuli J. Salmi, Lilja Jansson, Jyrki Launes, Laura Hokkanen, Anna-Kaisa Niemi, Kari Majamaa, Jari Lahti, Johan G. Eriksson, Timo Strandberg, Hannu Laaksovirta, Pentti J. Tienari

**Affiliations:** 1 Translational Immunology, Research Programs Unit, University of Helsinki, Helsinki, Finland; 2 Department of Neurology, Helsinki University Hospital, P.O. Box 63, 00014 Helsinki, Finland; 3 Department of Psychology and Logopedics, University of Helsinki, P.O. Box 21, 00014 Helsinki, Finland; 4 Research Unit of Clinical Neuroscience, Neurology, University of Oulu, P.O. Box 5000, 90014 Oulu, Finland; 5 Department of Neurology and Medical Research Center, Oulu University Hospital, Oulu, Finland; 6 Division of Neonatology, Rady Children’s Hospital San Diego, University of California San Diego, San Diego, CA USA; 7 Department of Obstetrics & Gynaecology and Human Potential Translational Research Programme, Yong Loo Lin School of Medicine, National University of Singapore and Singapore Institute for Clinical Sciences, Agency for Science, Technology and Research (A*STAR), Singapore, Singapore; 8 Department of General Practice and Primary Health Care, University of Helsinki and Helsinki University Hospital, Helsinki, Finland; 9 Folkhälsan Research Center, Helsinki, Finland; 10 Center for Life Course Health Research/Geriatrics, University of Oulu, Oulu, Finland; 11 Department of Medicine, Geriatric Clinic, University of Helsinki, Helsinki University Central Hospital, Helsinki, Finland

**Keywords:** ALS, *C9orf72*, Intermediate repeats, Case-control analysis, Aging

## Abstract

The hexanucleotide repeat expansion in intron 1 of the *C9orf72* gene causes amyotrophic lateral sclerosis (ALS) and frontotemporal dementia. In addition to the effects of the pathogenic expansion, a role of intermediate-length alleles has been suggested in ALS, corticobasal degeneration and Parkinson’s disease. Due to the rarity of intermediate-length alleles with over 20 repeats and the geographical variability in their frequency, large studies that account for population stratification are needed to elucidate their effects. To this aim, we used repeat-primed PCR and confirmatory PCR assays to determine the *C9orf72* repeat allele lengths in 705 ALS patients and 3958 controls from Finland. After exclusion of expansion carriers (25.5% of the ALS patients and 0.2% of the controls), we compared the frequency of intermediate-length allele carriers of 525 ALS cases and 3950 controls using several intermediate-length allele thresholds (7–45, 17–45, 21–45, 24–45 and 24–30). The carriership of an intermediate-length allele did not associate with ALS (Fisher’s test, all *p* ≥ 0.15) nor was there any association with survival (*p* ≥ 0.33), when we divided our control group into three age groups (18–65, 66–84 and 85–105 years). Carriership of two intermediate-length alleles was associated with ALS, when the longer allele was ≥ 17 repeats (*p* = 0.002, OR 5.32 95% CI 2.02–14.05) or ≥ 21 repeats (*p* = 0.00016, OR 15.21 95% CI 3.79–61.0). Our results show that intermediate-length alleles are a risk factor of ALS when present in both alleles, whereas carrying just one intermediate-length allele was not associated with ALS or survival.

**Electronic supplementary material:**

The online version of this article (10.1186/s40478-020-01059-5) contains supplementary material, which is available to authorized users.

## Introduction

The *C9orf72* hexanucleotide repeat expansion is the most common genetic cause of amyotrophic lateral sclerosis (ALS) and frontotemporal dementia (FTD) in populations of European descent [7, 16, 21]. Pathological expansions are usually hundreds to thousands of repeats in length [2, 25] and the expansion can exhibit somatic mosaicism [2]. The minimum pathogenic repeat length is unknown, but the threshold of 30 repeats has been commonly used. This threshold might be underestimated since we recently found that about 0.4% of aged Finns carried 30–45 repeat alleles and these individuals had no apparent increase in the frequency of neurodegenerative or psychiatric diseases [13].

There are contradictory reports on the effects of intermediate-length alleles under 30 repeats. For example, *C9orf72* intermediate-length alleles were reported to be associated with a higher risk of neuropsychiatric symptoms but not directly with various neurodegenerative diseases including ALS [18]. However, two recent large meta-analyses found an association between ALS and 24–30 repeat alleles [5, 12]. The latter study also showed a different frequency of the repeats between the northern and southern European populations [12]. The comparison of different studies on the effects of intermediate-length alleles is complicated by the varying intermediate-length allele thresholds. The different thresholds have been justified in many ways, e.g. association with the same risk haplotype as expansions (≥ 7 repeats) [24], effects on DNA methylation and gene expression (7–24 and 17–29 repeats) [3, 9] and associations with neurodegenerative diseases (e.g. 17–30, 20–22, 20–30, 24–30) [3, 10, 12, 20].

Here, we assessed the length of *C9orf72* hexanucleotide repeat alleles in 705 ALS patients and 3958 controls, tested their association with ALS and effect on survival in controls using several intermediate-length allele thresholds.

## Materials and methods

### Cohorts

We assessed the length of *C9orf72* hexanucleotide repeat alleles in 773 ALS cases, 405 of whom were included in a previous genome-wide association study and in the discovery study of *C9orf72* [15, 21]. The *C9orf72* repeat alleles were newly genotyped in 2019–2020 for all samples. There were 177 (23%) familial ALS cases and 596 apparently sporadic cases. The patients’ mean age-of-onset was 58 years and 51% were females. The patients were recruited at the Department of Neurology, Helsinki University Hospital, that receives referrals from neurologists throughout Finland. After exclusion of ALS cases with non-Finnish ancestry (n = 15) on the basis of patients’ self-reported birth-places and related individuals (n = 46, > 0.185 proportion identity-by-descent), there were 712 ALS cases remaining for the present study.

Our control group consisted of individuals from six cohorts. We have previously determined the *C9orf72* hexanucleotide repeat lengths in four cohorts and the cohort descriptions have been previously published [13]. In brief, the Vantaa 85+ study consists of 553 individuals of at least 85 years of age, who were living in the city of Vantaa, Finland on April 1st 1991 (DNA available in 486). The Helsinki Birth Cohort Study (HBCS) originally consisted of 8760 individuals born in Helsinki in 1934–1944, and a random subsample of 2003 individuals was neurologically assessed and underwent DNA extraction in 2001 (DNA was available in 1651 individuals for the present study). The Helsinki Businessmen study (HBS) included men with high socioeconomic status who were born in 1919–1934 and in 2002–2003, 672 individuals who lived at home were randomly selected for analyses (DNA was available in 666). The DEBATE study was originally a random sample of 4800 individuals from Helsinki out of whom 400 home-living individuals with stable cardiovascular disease were randomly selected for further studies in 2000 (DNA was available for 375). We excluded one individual diagnosed with ALS from these cohorts, which decreased the number of controls from the original 3142 to 3141.

The first of the new control cohorts is the PLASTICITY cohort [11], which consists of originally 1196 individuals born in 1971–74 in the Helsinki metropolitan area who had at least one predefined pre- or perinatal risk factor (e.g. birth weight ≤ 2000 g, Apgar score < 7, respiratory distress syndrome, diabetic mother, septic infection). At the age of 40 years, a survey using a mailed questionnaire about neurodevelopmental symptoms was conducted and 509 subjects responded (DNA available in 433). The second new control cohort includes 400 healthy blood donors aged 18–65 years at the time of study participation [17, 19], who were recruited in Northern Ostrobothnia (n = 102), Kainuu (n = 85), North Savo (n = 61), Pirkanmaa (n = 152) (DNA available in 400).

### Genotyping

We extracted DNA using standard methods from peripheral blood leukocytes (5 cohorts) or saliva (PLASTICITY cohort). We assessed the *C9orf72* hexanucleotide repeat alleles as previously described [21] with minor modifications [13]. In brief, for all samples, we first assessed repeat lengths using repeat-primed PCR (RP-PCR) followed by capillary electrophoresis and the results were visualized using the GeneMapper software v6 (ThermoFisher). Then, for samples with a putative expansion (including all samples with ≥ 20 repeats) or unclear zygosity, we performed over-the-repeat PCR. Samples that showed the typical saw tooth pattern in RP-PCR and did not produce longer amplicon in over-the-repeat PCR were categorized as expansions. The longest non-expanded (amplifiable) discrete allele we could detect was 45 repeats, and we used it as the expansion threshold [13]. Examples of RP-PCR capillary electrophoresis chromatograms and over-the-repeat PCR analyses are in Additional file 1: Supplementary Figure 1.

We used rs3849942 allele A as a marker for the *C9orf72* risk haplotype [2]. Samples from HBS (n = 669), PLASTICITY (n = 444) and DEBATE (n = 360) cohorts were genotyped with Illumina Global Screening Array 24v2-3, Vantaa85+ (n = 512) with Illumina HumanCNV370 array, HBCS (n = 1643) with Illumina 610k array and ALS cases (n = 803) with Affymetrix Axiom custom SNP array. Genotyping data underwent standard per-sample and per-variant quality control steps [1].

### Statistics

We used Fisher’s exact test to test for differences in the frequency of intermediate-length allele carriers between individuals with ALS and controls. We used the Cochran–Armitage trend test to estimate linear trends between the frequency of intermediate-length allele carriers and different age groups as implemented in the CATT package (Du and Hao 2017) in R version 3.5.2 (R Core Team 2017). Since there is no established threshold for intermediate-length alleles, in the main analysis we tested several thresholds with decreasing allele frequency: (1) 7–45 repeats, (2) 17–45 repeats, (3) 21–45 repeats, (4) 24–45 repeats and (5) 24–30 repeats. Since it can be assumed that the effect of the expansion overshadows any possible effects of an intermediate-length allele, we excluded all expansion carriers from the main analyses.

We calculated the statistical power of our study using the genpwr package (Moore, Jacobson 2019) as implemented in R version 3.5.2 (Additional file 1: Supplementary Table 1).

## Results

### *C9orf72* hexanucleotide repeat expansion and intermediate-length alleles in the Finnish cohorts

We successfully genotyped 705/712 (99.0%) of the ALS patients and 3958/3993 (99.1%) of the controls. There were 180 (25.5%) ALS cases and 8 (0.20%) controls who carried the *C9orf72* hexanucleotide repeat expansion and they were excluded from the main analyses leaving 525 ALS cases and 3950 controls.

The demographic details and *C9orf72* intermediate-length allele and expansion carrier frequencies as well as *C9orf72* risk haplotype tagging SNP frequency in each control cohort are in Additional file 1: Supplementary Table 2.

### Association of *C9orf72* hexanucleotide repeat intermediate-length alleles with ALS

We did not observe any significant differences in the frequencies of the intermediate-length allele carriers between ALS patients and controls (all *p* ≥ 0.15) (Table 1). The results did not markedly change when the 188 individuals with an expansion were included (all *p* ≥ 0.31, Additional file 1: Supplementary Table 3). All intermediate-length allele carrier frequencies (without predefined thresholds) in cases and controls are presented in Additional file 1: Supplementary Table 4.

**Table 1:** *C9orf72* hexanucleotide repeat intermediate-length allele carriers in 525 ALS patients and 3950 controls after exclusion of expansion carriers

Longer allele	Controls n	ALS n	*p*	OR	95% CI
7–45	1293 (33%)	185 (35%)	0.26	1.12	0.92–1.36
17–45	101 (2.6%)	19 (3.6%)	0.15	1.43	0.82–2.74
21–45	66 (1.7%)	12 (2.3%)	0.29	1.38	0.67–2.59
24–45	43 (1.1%)	6 (1.1%)	0.82	1.05	0.36–2.50
24–30	30 (0.76%)	3 (0.57%)	1	0.75	0.15–2.43

We made an additional analysis in individuals with two intermediate-length alleles. There was a statistically significant difference in the frequency of individuals with any two copies of intermediate-length alleles (7–45 repeats) between ALS (26/525, 5.0%) and controls (104/3950, 2.6%) (*p* = 0.005, OR = 1.93 [1.19–3.02]). Table 2 illustrates the risk of ALS in individuals with two copies of the intermediate-length alleles stratified by the length of the longer allele. In this analysis the increased risk is observed when the longer allele is ≥ 17 repeats (Table 2). Our data is not powered to pinpoint an exact threshold of increased risk, all genotypes of the cases and controls with two copies of intermediate-length alleles are shown in Additional file 1: Supplementary Table 5. Most individuals with two intermediate-length alleles were compound heterozygotes, i.e. they had two different intermediate-length alleles. (62% of ALS cases and 66% of the controls, Additional file 1: Supplementary Table 5).

**Table 2:** Individuals with two intermediate-length alleles in 525 ALS patients and 3950 controls after exclusion of expansion carriers

Shorter/longer allele	Controls n (%)	ALS n (%)	*p*	OR	95% CI
≥ 7/≥ 7	104 (2.6%)	26 (5.0%)	0.005	1.93	1.19–3.02
7–16/7–16	94 (2.4%)	19 (3.6%)	0.098	1.57	0.90– 2.62
≥ 7/17–45	10 (0.25%)	7 (1.3%)	0.0020	5.32	1.71–15.56
≥ 7/21–45	3 (0.076%)	6 (1.1%)	0.00016	15.19	3.23–94.21
≥ 7/24–45	1 (0.025%)	4 (0.76%)	0.00085	30.26	2.99–1479

### *C9orf72* risk haplotype

The haplotype tagging SNP rs3849942 was successfully genotyped in 683 ALS cases and 3178 controls. After the exclusion of expansion carriers, the frequency of rs3849942 (A) carriers (marker of the risk haplotype) was 995/3172 (31.4%) in the controls and 182/505 (36.0%) in ALS cases (*p* = 0.041, OR = 1.23 [1.01–1.50], χ2 test). The difference was largely driven by the higher number of individuals homozygous for the rs3849942 (A) allele in ALS cases than in controls (Table 3). The odds ratio of homozygosity for rs3849942 (A) was 1.89 (95% CI 1.20–2.99, *p* = 0.018). This association was lost, when individuals with intermediate-length alleles were excluded as there were one ALS case and four controls who had rs3849942 A/A genotype but no intermediate-length allele (*p* = 0.51 Fisher’s test). Additional file 1: Supplementary Table 4 shows the distribution of rs3849942 (A) in carriers of each intermediate-length allele in ALS patients and controls. Practically all intermediate-length allele carriers with ≥ 17 repeats were also carriers of rs3849942 (A) in ALS cases and controls (with one exception among the 76 controls with ≥ 17 repeats and rs3849942 data available).

**Table 3:** Comparison of rs3849942 genotypes in 505 ALS cases and 3172 controls after the exclusion of expansion carriers

rs3849942 genotype	Controls n (%)	ALS n (%)	χ^2^
G/G	2177 (68.6%)	323 (64.0%)	1.40
G/A	910 (28.7%)	157 (31%)	0.87
A/A	85 (2.7%)	25 (5.0%)	7.51
Total	3172	505	9.77, 2df, *p* = 0.0076

### *C9orf72* hexanucleotide repeat intermediate-length allele distributions in different age groups

To test for a possible effect on survival presenting as a difference in the frequency of intermediate-length allele carriers in different age groups, we divided the controls into three age categories 18–65, 66–84 and 85–106 years. We did not observe a statistically significant linear trend between the proportion of intermediate-length allele carriers and age groups (Table 4).

**Table 4:** Intermediate repeat length allele frequencies in different age groups of controls

	18–65 years	66–84 years	85–105 years	*p* value^a^
Longer allele				
7–45	273 (31%)	656 (33%)	363 (33%)	0.46
17–45	29 (3.3%)	44 (2.2%)	28 (2.5%)	0.33
21–45	18 (2.1%)	29 (1.5%)	19 (1.7%)	0.62
24–45	12 (1.4%)	17 (0.86%)	14 (1.3%)	0.92
24–30	10 (1.1%)	9 (0.46%)	11 (1.0%)	0.85
Two 7–45 alleles	26 (3.0%)	51 (2.6%)	27 (2.5%)	0.48
Longer allele in individuals with two 7–45 alleles				
7–16	23 (2.6%)	47 (2.4%)	24 (2.2%)	
≥ 17	3 (0.34%)	4 (0.20%)	3 (0.27%)	
≥ 21	1 (0.11%)	2 (0.10%)	0	
≥ 24	0	1 (0.050%)	0	
N of age group	873	1973	1102	

^a^Cochran–Armitage trend test. Excluded 10 individuals from whom age group was unknown

We did not have the power to detect differences in carriers of two copies of intermediate-length alleles due to their rarity but analyzed them for descriptive purposes. This analysis showed that in the oldest age group (≥ 85 years) there were no individuals with two intermediate-length alleles where the longer allele was ≥ 21 repeats. The *C9orf72* haplotype tagging SNP rs3849942 homozygosity (A/A) was found in 16/436 (3.7%), 43/1688 (2.5%) and 26/1047 (2.5%) in the age groups 18–65, 66–84 and ≥ 85 years respectively (*p* = ,0.28 Cochran–Armitage trend test).

## Discussion

In our analyses, the frequencies of the *C9orf72* hexanucleotide repeat intermediate-length allele carriers did not show a statistically significant difference between Finnish ALS patients and controls when only the longer allele was considered (all *p* ≥ 0.15). In addition, we did not observe any significant differences in the frequency of carriers of the intermediate-length alleles in controls belonging to different age groups (Table 3) suggesting that carriership of these alleles are not subject to strong negative selection during aging. Hence, just carriership of an intermediate-length allele does not confer increased risk of ALS or play a major role in survival in the Finnish population.

However, when individuals with two copies of the intermediate-length alleles were studied, significant associations were found, especially when the longer allele was ≥ 17 repeats (Table 2). The highest homozygous number of repeats was 12 repeats (Additional file 1: Supplementary Table 5) indicating that most of the risk genotypes were compound heterozygotes. Compound heterozygote effect of the intermediate-length CAG-repeat alleles has been previously shown in spinocerebellar ataxia 3 [22]. The “two copy effect” was further supported by the association of the risk haplotype marker homozygosity with ALS (Table 3).

Our results suggest that in the Finnish population, carrying one intermediate-length allele does not significantly increase the risk of ALS but having two such alleles and one of these ≥ 17 repeats raises the odds of ALS about fivefold. This is not a big increase in risk in a rare disease like ALS with a lifetime risk of ca. 1/400. Therefore, in ALS patients with two intermediate-length alleles, the effect of additional genetic or environmental factors is likely important for the disease development. It is of note, that one of our ALS patients who had 8/36 repeat alleles was also homozygous for the SOD1*D91A mutation and survived 14 years after diagnosis. Such a long survival is typical in SOD1*D91A ALS but not in *C9orf72* ALS suggesting that the major genetic factor modulating the clinical picture was the *SOD1* mutation.

Our results agree with a previous study that reported in Belgian ALS and FTD–ALS patients (n = 135) a significantly increased risk in carriers of two copies of the intermediate-length alleles (OR 2.08, *p* = 0.04) [9]. Also, in a Flanders–Belgian cohort of patients with FTD (without *C9orf72* expansion) a significant risk for homozygous carriers of the *C9orf72* risk haplotype tagging SNP was found (OR 1.75, *p* = 0.04), which disappeared after removal of intermediate-length allele carriers [24]. Functionally the intermediate-length alleles have been shown to modulate expression of *C9orf72*, which may vary in different tissues [3, 9]. In nerve tissue the intermediate-length alleles and the risk haplotype tagging SNP have been shown to result in increased *C9orf72* transcription from the promoter upstream of the hexanucleotide repeat, while intermediate alleles did not generate RNA foci or dipeptide repeat proteins that are pathognomonic for the *C9orf72* expansion [3].

Our results are somewhat in contrast with a recent meta-analysis comprising 5071 ALS cases and 3747 controls that reported an association of 24-30 repeat alleles with ALS with an odds ratio 4.20 (random-effects model *p* = 0.02) [12]. The association with 24–30 repeats was robust and the association was observed even when omitting one study at a time in the meta-analysis. Another report suggesting the pathogenicity of alleles of ≥ 17 repeats studied autopsy-proven corticobasal degeneration cases (CBD) from UK and USA versus global controls [3]. These intermediate-length alleles also increased *C9orf72* expression in the human brain and in neural progenitor cells [3]. Complete genotypes were not reported in the ALS meta-analysis, while in the CBD study 2 out of 13 cases were compound heterozygotes for an intermediate-length allele. In both studies, it was assumed that carriership of either 24–30 repeats (ALS) or ≥ 17 repeats (CBD) was the main risk factor for disease. Our data does not support this assumption to be true in Finland. There are several potential explanations for our different results.

First, the genetics and penetrance of the intermediate-length alleles might be different in Finland versus other populations. All carriers of intermediate-length alleles of ≥ 17 repeats were also carriers of the risk haplotype-tagging SNP (with only one exception, Additional file 1: Supplementary Table 5), which indicates that the gross haplotype background is similar in Finland and other populations. However, there could be a protective factor in Finland that decreases the risk of heterozygotes and allows the carrier frequencies to rise in normal population. It is of note that two copies of the intermediate-length alleles were required for increased risk in Belgian ALS and FTD-ALS patients, too [9]. Hence, the hypothetical protective factor should be widely spread in Europe.

**Fig. 1:**
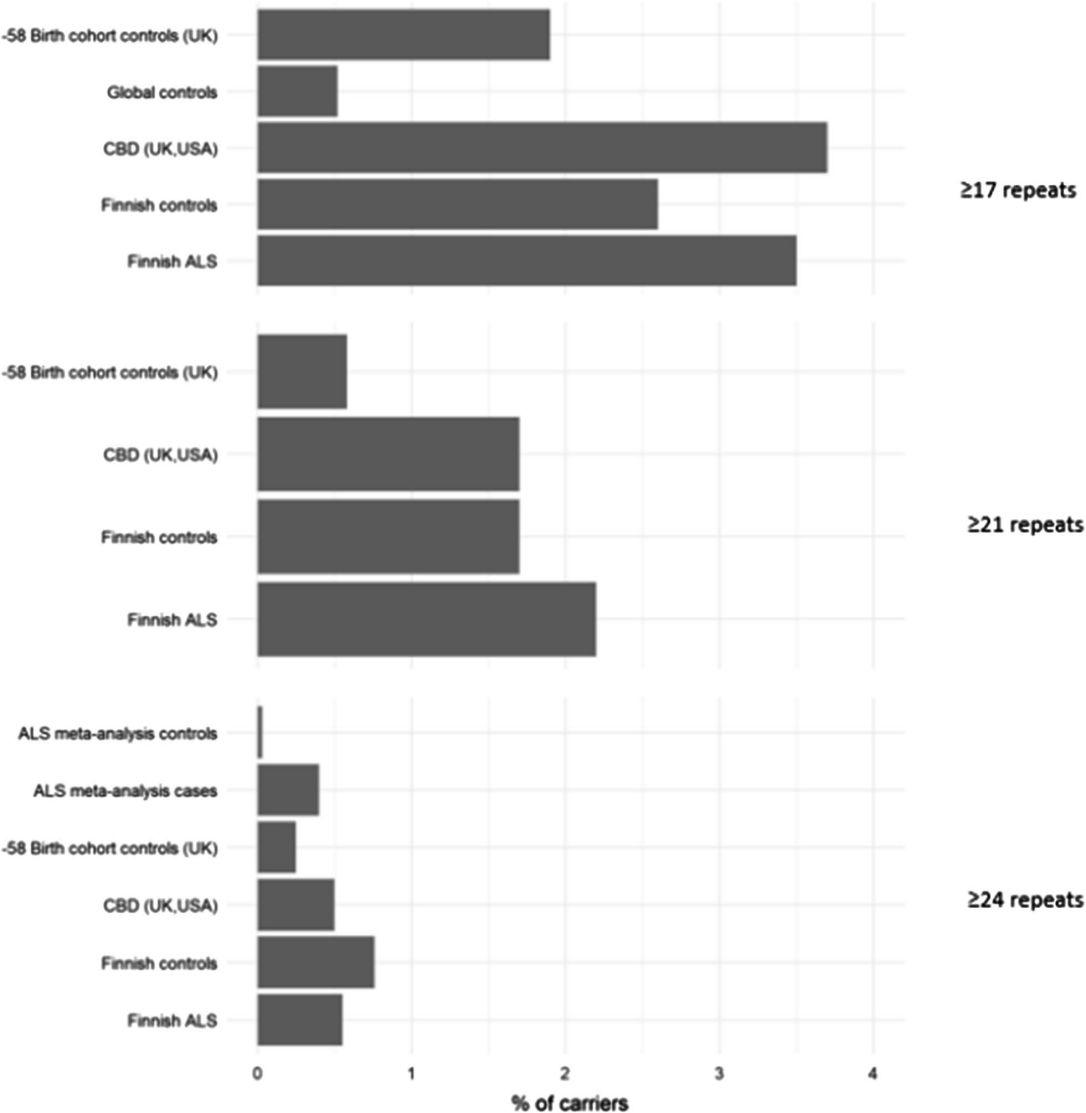
The frequencies of non-expanded long *C9orf72* hexanucleotide repeat intermediate-length alleles in three previous case-control studies in comparison with Finnish ALS cases and controls. ≥ 17 repeat alleles were found in 3.6% of the Finnish ALS cases, 2.6% of the controls (present study) and in 3.7% of cases with autopsy-proven corticobasal degeneration (CBD) as compared to 0.52% of the Global controls [3] and 1.9% for UK 58 Birth cohort [2]. The majority of cases were from three centers from USA and UK, while the controls were from a global study [23]. Allele frequencies for 58BC were obtained as personal written communication from Professor Simon Mead. Each ≥ 17 allele is presumed to be carried by one individual. ≥ 21 repeat alleles were found in 2.3% of the Finnish ALS cases and 1.7% of the controls (present study), and in 1.7% of CBD cases. These alleles were not reported in the Global controls [23]. 24–30 repeat alleles were found in 0.57% of the Finnish ALS cases and 0.76% of controls (present study),and in 0.5% of CBD cases and in 0.4% of ALS cases vs. 0.03% of controls in a recent meta-analysis [10]

Second, population stratification can confound the analyses. The observed ethnic and geographic differences in the intermediate-length allele frequencies [12, 13] may potentially result in spurious associations due to population stratification. The discrepancy between our results and previous reports seem to be largely driven by the higher frequency of the long intermediate-length alleles (≥ 20 repeats) in our Finnish controls. The frequencies of the long intermediate-length alleles in ALS and CBD cases show much less dispersion than controls between populations as illustrated in Fig. 1. Therefore, studies using multinational cohorts need to carefully account for population stratification and, additionally, use sufficiently large subcohorts to ensure that rare alleles can be captured.

Third, different genotyping methodology of the *C9orf72* hexanucleotide repeat can affect results. It is well-known that genotyping of the *C9orf72* hexanucleotide repeat can be challenging, as the large size and high GC content of the *C9orf72* expansion does not allow full polymerization during PCR. Even accredited laboratories report different genotypes from the same sample [6]. For differentiating homozygotes and heterozygotes in unclear samples, we used an additional confirmatory PCR method (Additional file 1: Supplementary Figure 1). There may also be slight inconsistencies in the determination of the exact lengths of the longer alleles (e.g. 23 vs. 24 repeats) with RP-PCR. For this reason in our analyses the smallest range of repeat length was seven (24–30 repeats). Meta-analyses rely on previous studies often with varying genotyping methodologies, which can make the harmonization of data difficult. The different sources of DNA is another possible confounding factor, as the use of blood versus brain or lymphoblastoid cell line derived DNA may cause significant bias [2, 8] although not yet systematically studied in *C9orf72* intermediate-length alleles. Huntington’s disease CAG-repeat represents a case in which the size of an intermediate allele with 34 repeats has been shown to increase in serially passaged lymphoblastoid cell lines [4].

Our study’s strength is the focus on Finnish individuals, which decreases the effects of population stratification and allows a sufficiently large number of longer intermediate-length allele carriers for comparisons because these alleles are relatively common in this population [13]. However, it also affects the generalizability of our results. Based on our assessment of *C9orf72* repeat length Finns have more 2–6 repeat alleles, less 7–19 repeat alleles and again more ≥ 20 repeat alleles than the general European population. The risk haplotype (and intermediate-length allele) tagging rs3849942 (A) is less common in Finland as compared to non-Finnish Europeans and reflects the lower frequency of the 7–19 repeat alleles in Finns. Therefore, we cannot exclude the possibility of unknown selection factors facilitating the enrichment of ≥ 20 alleles in the Finnish population.

Our study has also limitations. The controls and ALS cases were genotyped for rs3849942 using different platforms and different genotyping centers, which can lead to slight differences in allele frequencies. However, we do not believe this to be an important source of error, since the allele frequencies in our controls were consistent with the Finnish samples of the gnomAD database v.3 [14]. Moreover, we had whole-genome sequencing data on ca. 300 ALS cases that had also been genotyped for rs3849942 and the concordance of genotypes was 100%. Furthermore, RP-PCR, which we used to assess *C9orf72* repeat lengths, is accurate in detecting expansions and sizing most alleles, but like with other genotyping methods, the longer the allele, the harder it is to assess the exact length. Given that rs3849942 genotypes and the *C9orf72* repeat lengths correlated well, the number of wrongly assessed genotypes is likely small, and should not affect the conclusions of the study. We genotyped all cohorts except one from peripheral blood-derived DNA and age of the DNA samples varied. The cohort with saliva-derived DNA had the lowest genotyping success rate (97.2% vs. 98.9–99.4% in other cohorts), but the genotype distribution did not differ significantly from the other cohorts suggesting that the different source of DNA should not cause meaningful bias to the results. On the other hand, although we did not observe any marked difference in the *C9orf72* allele distribution of our younger and older control individuals, we cannot exclude small effects on survival, undetectable with the present sample size. A larger study is required to analyze small effects on survival and to test also survival in carriers of two copies of intermediate-length alleles and homozygotes for the C9orf72 risk haplotype tagging SNPs.

## Conclusions

Our data indicate that the *C9orf72* intermediate-length alleles increase the risk of ALS when present in two copies, carriership of only one intermediate-length allele is not associated with ALS or survival in the Finnish population. The clinical significance of the intermediate-length alleles should be critically re-assessed in carriers of one and two copies of these alleles.

## **Supplementary information**

**Additional file 1** Power calculations and detailed genotyping information.

## Data Availability

The datasets used and/or analysed during the current study available from the corresponding author on reasonable request.

## Abbreviations

ALS: amyotrophic lateral sclerosis
CBD: corticobasal degeneration
CI 95%: 95% confidence interval
FTD: frontotemporal dementia
HBS: Helsinki Businessmen study
HBSC: Helsinki Birth cohort study
MAF: minor allele frequency
OR: odds ratio
RP-PCR: repeat-primed PCR
