# Supplementary material for: Carriership of two copies of C9orf72 hexanucleotide repeat intermediate-length alleles is a risk factor for ALS in the Finnish population
Source: Acta Neuropathol Commun. 2020 Nov 9;8:187. doi: 10.1186/s40478-020-01059-5 (PMC7654028; doi:10.1186/s40478-020-01059-5)
Supplement: Supplementary file 1 — Additional file 1 Power calculations and detailed genotyping information. [file 40478_2020_1059_MOESM1_ESM.docx]

SUPPLEMENTARY DATA

***Carriership of two copies of C9orf72 hexanucleotide repeat intermediate-length alleles***

***is a risk factor for ALS in the Finnish population***

Karri Kaivola^1,2^, Samuli J. Salmi^1,2^, Lilja Jansson^1,2^, Jyrki Launes^3^, Laura Hokkanen^3^, Anna-Kaisa Niemi^4,5,6^, Kari Majamaa^4,5^, Jari Lahti^3^, Johan G. Eriksson^7,8,9^, Timo Strandberg^10,11^, Hannu Laaksovirta^1,2^*, Pentti J. Tienari^1,2^*

*Equal contribution

1. Translational Immunology, Research Programs Unit, University of Helsinki, Helsinki, Finland

2. Department of Neurology, Helsinki University Hospital, P.O. Box 63, 00014, Helsinki, Finland

3. Department of Psychology and Logopedics, University of Helsinki, P.O. Box 21, 00014, Helsinki, Finland.

4. Research Unit of Clinical Neuroscience, Neurology, University of Oulu, P.O. Box 5000, FI-90014, Oulu, Finland.

5. Department of Neurology and Medical Research Center, Oulu University Hospital, Oulu, Finland.

6. Division of Neonatology, Rady Children's Hospital San Diego, University of California San Diego, San Diego, California, USA

7. Department of Obstetrics & Gynaecology and Human Potential Translational Research ProgrammeYong Loo Lin School of Medicine, National University of Singapore and Singapore Institute for Clinical Sciences, Agency for Science, Technology and Research (A*STAR) SingaporeSingapore
8. Department of General Practice and Primary Health Care, University of Helsinki and Helsinki University Hospital, Helsinki, Finland.

9. Folkhälsan Research Center, Helsinki, Finland

10. Center for Life Course Health Research/Geriatrics, University of Oulu, Oulu, Finland

11. Department of Medicine, Geriatric Clinic, University of Helsinki, Helsinki University Central Hospital, Helsinki, Finland

**Power calculations**

We calculated statistical power and detectable effect sizes (odds ratios) using the genpwr package (Moore, Jacobson, 2019) as implemented in R version 3.5.2. Since we compared carrier frequencies, we used dominant model as the test model and dominant and additive models as the true models. The total sample size was approximately 4500 and case rate 12%. Alpha was 0.05 and power 80%. Minor allele frequencies (MAF) of the intermediate repeat length alleles were derived from the control population.

**Supplementary Table 1: Estimates of detectable effect size (odds ratios) after exclusion of expansion carriers**

| **Test Model** | **True Model** | **MAF** | **Power** | **N total** | **N cases** | **N Controls** | **Case rate** | **OR at α=0.05** |
| --- | --- | --- | --- | --- | --- | --- | --- | --- |
| Dominant | Dominant | 0.17 | 0.8 | 4475 | 537 | 3938 | 0.12 | 1.31 |
| Dominant | Additive | 0.17 | 0.8 | 4475 | 537 | 3938 | 0.12 | 1.28 |
| Dominant | Dominant | 0.013 | 0.8 | 4475 | 537 | 3938 | 0.12 | 2.01 |
| Dominant | Additive | 0.013 | 0.8 | 4475 | 537 | 3938 | 0.12 | 2.00 |
| Dominant | Dominant | 0.0083 | 0.8 | 4475 | 537 | 3938 | 0.12 | 2.31 |
| Dominant | Additive | 0.0083 | 0.8 | 4475 | 537 | 3938 | 0.12 | 2.30 |
| Dominant | Dominant | 0.0054 | 0.8 | 4475 | 537 | 3938 | 0.12 | 2.72 |
| Dominant | Additive | 0.0054 | 0.8 | 4475 | 537 | 3938 | 0.12 | 2.71 |
| Dominant | Dominant | 0.0038 | 0.8 | 4475 | 537 | 3938 | 0.12 | 3.18 |
| Dominant | Additive | 0.0038 | 0.8 | 4475 | 537 | 3938 | 0.12 | 3.17 |

**Supplementary Table 2: Demographic details and C9orf72 hexanucleotide repeat intermediate length allele frequencies in each control subcohort (expansion carriers included).**

|  | HBCS | HBS | Vantaa85+ | Debate | Plasticity | Blood donors |
| --- | --- | --- | --- | --- | --- | --- |
| Number of successfully genotyped individuals | 1643 | 640 | 486 | 372 | 421 | 396 |
| Mean age^a^ (range) | 71 (58-79) | 84 (60-95) | 91 (85-105) | 88 (75-101) | 42 (39-45) | 41 (18-65) |
| Percentage females | 56 % | 0 % | 80 % | 65 % | 52 % | 40 % |
| 7-45 repeats | 545 (33%) | 220 (34%) | 150 (31%) | 120 (32%) | 150 (36%) | 108 (28%) |
| 17-45 repeats | 35 (2.1%) | 18 (2.8%) | 15 (3.1%) | 6 (1.6%) | 12 (2.8%) | 15 (3.8%) |
| 21-45 repeats | 27 (1.6%) | 10 (1.6%) | 10 (2.1%) | 3 (0.81%) | 6 (1.4%) | 10 (2.5%) |
| 24-45 repeats | 16 (0.97%) | 8 (1.3%) | 7 (1.4%) | 2 (0.54%) | 3 (0.69%) | 7 (1.5%) |
| 24-30 repeats | 10 (0.61%) | 4 (0.63%) | 6 (1.2%) | 2 (0.54)% | 2 (0.48%) | 6 (1.5%) |
| Expansions | 6 (0.37%) | 0 | 0 | 0 | 1 (0.23%) | 1 (0.25%) |
| rs3849942 (A) AF in  genotyped samples  (n minor/total alleles) | 16.8%  (462/2742) | 17.1%  (210/1228) | 15.7%  (147/938) | 17.5%  (118/674) | 19.3%  (149/774) | Not genotyped |

^a^ Age at last examination, except in Vantaa85+, where age refers to age at death. The frequency of intermediate repeat length carriers between the subcohorts did not show statistically significant difference (Fisher’s/Chi^2^ test p≥ 0.069 in all intermediate length allele categories using 2x6 contingency tables). Smallest pairwise p = 0.011 (Plasticity vs. Blood donors 7-45 repeats). rs3849942 was successfully genotyped in >99% in all cohorts. AF: allele frequency.

**Supplementary Figure 1. Genotype verificiation on gel. A. Example RP-PCR capillary electrophoresis chromatograms. B. Example of over-the-repeat PCR gel verification** **for homo-/heterozygosity and bi- vs. monoallelic amplification. Expansion results in monoallelic amplification in over-the-repeat PCR. (N.B. 2 and 3 repeat alleles cannot be separated, allele 2 denotes alleles 2/3)**

**A.**
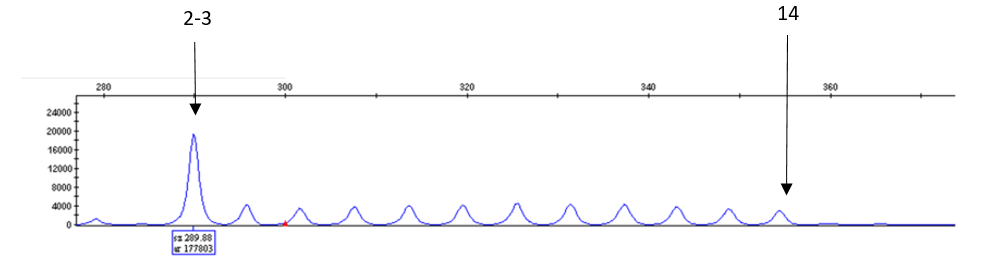


*First peak corresponds to 2-3 repeats.


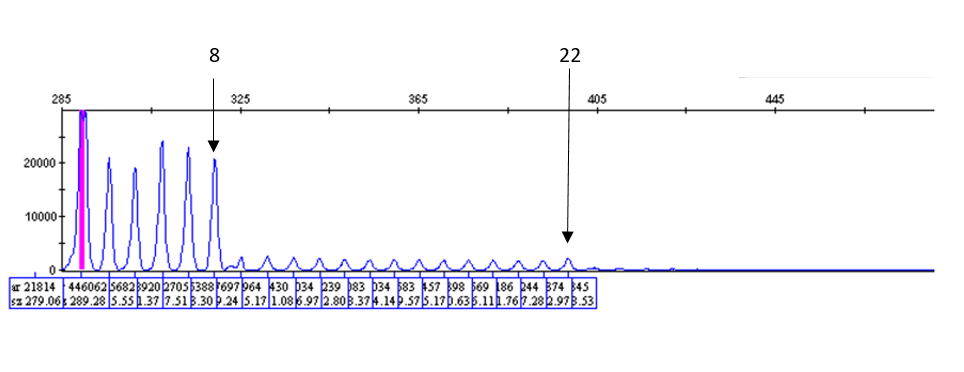


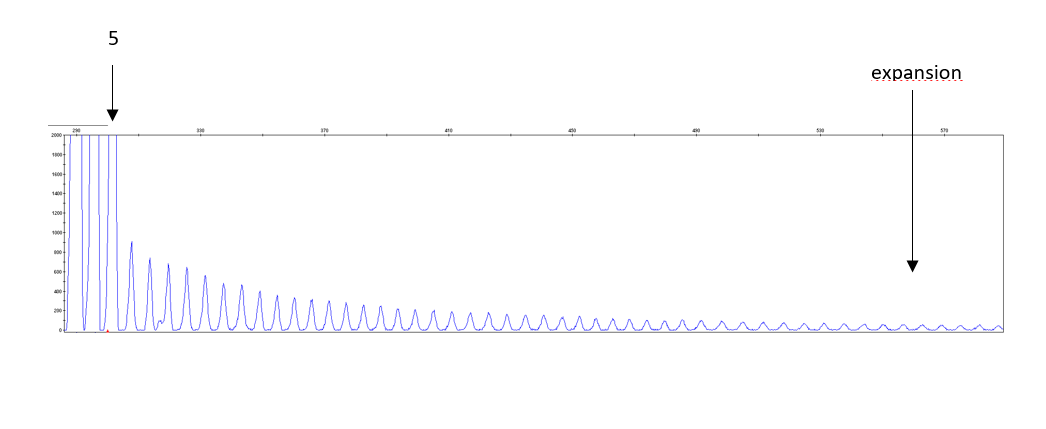


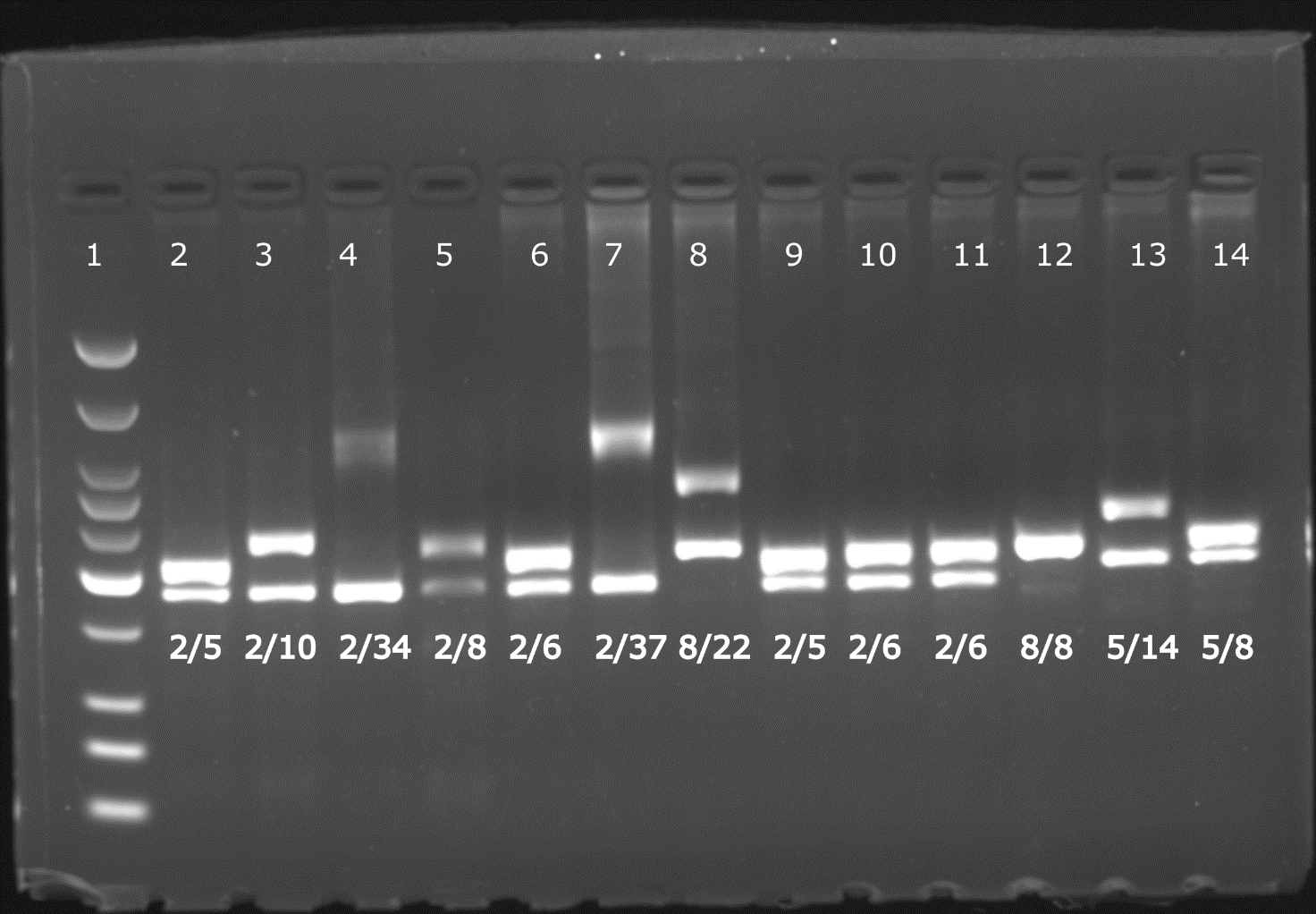
**B.**


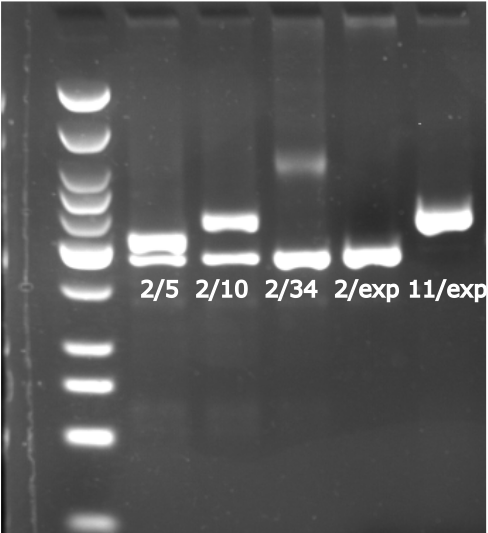


**Supplementary Table 3: *C9orf72* intermediate-length allele carriers in 705 ALS patients and 3 958 controls including expansion carriers.**

| **Longer allele*** | **n (%) controls** | **n (%) ALS** | **p (Fisher)** | **OR** | **CI 95%** |
| --- | --- | --- | --- | --- | --- |
| 7-45 | 1294 (33%) | 222 (31%) | 0.54 | 0.95 | 0.79-1.13 |
| 17-45 | 101 (2.6%) | 23 (3.3%) | 0.31 | 1.29 | 0.78-2.06 |
| 21-45 | 66 (1.7%) | 15 (2.1%) | 0.43 | 1.28 | 0.68-2.29 |
| 24-45 | 43 (1.1%) | 8 (1.1%) | 0.85 | 1.05 | 0.42-2.26 |
| 24-30 | 30 (0.76%) | 4 (0.57%) | 0.81 | 0.74 | 0.19-2.13 |
| Expansion | 8 | 180 | 1.08E-144 | 169 | 83-397 |

Of the expansion carriers 37/185 ALS cases (20%) and 1/8 control (13%) had an intermediate-length allele (≥7) and the expansion. *Length of the non-expanded allele in expansion carriers.

**Supplementary Table 4. Analysis of the C9orf72 intermediate-length allele carriers using discrete values in controls and ALS cases and the proportion of rs3849942A carriers.**

| **Longer allele length** | **n (%*) Controls** | **n (%) ALS** | **n rs3849942 data Controls** | **n (%**) rs3849942 (A) carriers controls** | **n rs3849942 data ALS** | **n (%**) rs3849942 (A) carriers ALS** |
| --- | --- | --- | --- | --- | --- | --- |
| 2-3 | 1358 (34.4%) | 173 (33.0%) | 1084 | 11 (1.0%) | 167 | 4 (2.4%) |
| 4 | 77 (1.9%) | 7 (1.3%) | 66 | 2 (3.0%) | 7 | 0 (0%) |
| 5 | 1012 (25.6%) | 127 (24.2%) | 813 | 62 (7.6%) | 122 | 14 (11.5%) |
| 6 | 210 (5.3%) | 33 (6.3%) | 157 | 8 (5.1%) | 32 | 2 (6.3%) |
| 7 | 118 (2.99%) | 10 (1.90%) | 93 | 21 (23%) | 10 | 4 (40%) |
| 8 | 665 (16.84%) | 101 (19.24%) | 543 | 492 (91%) | 97 | 88 (91%) |
| 9 | 23 (0.58%) | 2 (0.38%) | 20 | 16 (80%) | 1 | 1 (100%) |
| 10 | 203 (5.14%) | 31 (5.90%) | 171 | 164 (96%) | 31 | 31 (100%) |
| 11 | 41 (1.04%) | 6 (1.14%) | 31 | 30 (97%) | 5 | 5 (100%) |
| 12 | 38 (0.96%) | 5 (0.95%) | 31 | 30 (97%) | 5 | 5 (100%) |
| 13 | 27 (0.68%) | 3 (0.57%) | 22 | 22 (100%) | 3 | 3 (100%) |
| 14 | 38 (0.96%) | 5 (0.95%) | 32 | 32 (100%) | 4 | 4 (100%) |
| 15 | 20 (0.51%) | 1 (0.19%) | 15 | 13 (87%) | 1 | 1 (100%) |
| 16 | 19 (0.48%) | 2 (0.38%) | 18 | 17 (94%) | 2 | 2 (100%) |
| 17 | 11 (0.28%) | 1 (0.19%) | 9 | 9 (100%) | 1 | 1 (100%) |
| 18 | 10 (0.25%) | 3 (0.57%) | 7 | 7 (100%) | 3 | 3 (100%) |
| 19 | 6 (0.15%) | 3 (0.57%) | 3 | 3 (100%) | 3 | 3 (100%) |
| 20 | 8 (0.20%) | 0 | 6 | 6 (100%) | 0 | 0 |
| 21 | 4 (0.10%) | 2 (0.38%) | 4 | 4 (100%) | 1 | 1 (100%) |
| 22 | 12 (0.30%) | 3 (0.57%) | 10 | 10 (100%) | 3 | 3 (100%) |
| 23 | 7 (0.18%) | 1 (0.19%) | 6 | 6 (100%) | 1 | 1 (100%) |
| 24 | 6 (0.15%) | 2 (0.38%) | 4 | 4 (100%) | 2 | 2 (100%) |
| 25 | 8 (0.20%) | 1 (0.19%) | 6 | 6 (100%) | 1 | 1 (100%) |
| 26 | 4 (0.10%) | 0 | 2 | 2 (100%) | 0 | 0 |
| 27 | 5 (0.13%) | 0 | 4 | 4 (100%) | 0 | 0 |
| 28 | 1 (0.03%) | 0 | 0 | 0 | 0 | 0 |
| 29 | 4 (0.10%) | 0 | 3 | 3 (100%) | 0 | 0 |
| 30 | 2 (0.051%) | 0 | 1 | 1 (100%) | 0 | 0 |
| 31 | 2 (0.051%) | 0 | 2 | 2 (100%) | 0 | 0 |
| 32 | 3 (0.076%) | 0 | 3 | 3 (100%) | 0 | 0 |
| 33 | 0 (0%) | 0 | 0 | 0 | 0 | 0 |
| 34 | 1 (0.025%) | 0 | 1 | 1 (100%) | 0 | 0 |
| 35 | 0 (0%) | 0 | 0 | 0 | 0 | 0 |
| 36 | 2 (0.051%) | 1 (0.19%) | 1 | 0 (0%) | 1 | 1 (100%) |
| 37 | 0 (0%) | 2 (0.38%) | 0 | 0 | 2 | 2 (100%) |
| 38 | 0 (0%) | 0 | 0 | 0 | 0 | 0 |
| 39 | 0 (0%) | 0 | 0 | 0 | 0 | 0 |
| 40 | 1 (0.025%) | 0 | 1 | 1 (100%) | 0 | 0 |
| 41 | 0 (0%) | 0 | 0 | 0 | 0 | 0 |
| 42 | 0 (0%) | 0 | 0 | 0 | 0 | 0 |
| 43 | 1 (0.025%) | 0 | 1 | 1 (100%) | 0 | 0 |
| 44 | 0 (0%) | 0 | 0 | 0 | 0 | 0 |
| 45 | 3 (0.076%) | 0 | 2 | 2 (100%) | 0 | 0 |
| Total | 3950 | 525 | 3172 | 995 | 505 | 180 |

*Percentage calculated from total amount of controls and cases when expansion carriers are excluded.
** Percentage from successfully genotyped samples

**Supplementary Table 5: Genotypes of individuals homozygous for intermediate-length (7-45) alleles.**

| Allele1 | Allele2 | n in ALS  n=525 | n in Controls  n=3950 |
| --- | --- | --- | --- |
| 7 | 7 | 1 (0.2%) | 0 |
| 7 | 8 | 0 | 8 (0.2%) |
| 7 | 10 | 2 (0.4%) | 5 (0.1%) |
| 7 | 11 | 0 | 1 (0.03%) |
| 7 | 12 | 1 (0.2%) | 0 |
| 7 | 13 | 0 | 1 (0.03%) |
| 7 | 14 | 0 | 2 (0.05%) |
| 7 | 15 | 0 | 1 (0.03%) |
| 7 | 16 | 1 | 0 |
| 7 | 24 | 1 (0.2%) | 0 |
| 8 | 8 | 7 (1.3%) | 26 (0.7%) |
| 8 | 9 | 0 | 2 (0.05%) |
| 8 | 10 | 2 (0.4%) | 14 (0.4%) |
| 8 | 11 | 0 | 4 (0.1%) |
| 8 | 12 | 0 | 4 (0.1%) |
| 8 | 13 | 1 (0.2%) | 2 (0.05%) |
| 8 | 14 | 0 | 5 (0.1%) |
| 8 | 15 | 0 | 5 (0.1%) |
| 8 | 16 | 0 | 1 (0.03%) |
| 8 | 18 | 0 | 2 (0.05%) |
| 8 | 20 | 0 | 1 (0.03%) |
| 8 | 22 | 2 (0.4%) | 2 (0.05%) |
| 8 | 24 | 1 (0.2%) | 0 |
| 8 | 36 | 1 (0.2%) | 0 |
| 8 | 45 | 0 | 1 (0.03%) |
| 9 | 12 | 1 (0.2%) | 0 |
| 10 | 10 | 0 | 7 (0.2%) |
| 10 | 11 | 0 | 1 (0.03%) |
| 10 | 13 | 0 | 1 (0.03%) |
| 10 | 14 | 0 | 1 (0.03%) |
| 10 | 16 | 0 | 1 (0.03%) |
| 10 | 17 | 0 | 2 (0.05%) |
| 10 | 18 | 1 (0.2%) | 1 (0.03%) |
| 10 | 20 | 0 | 1 (0.03%) |
| 11 | 11 | 1 (0.2%) | 0 |
| 12 | 12 | 1 (0.2%) | 2 (0.05%) |
| 12 | 14 | 1 (0.2%) | 0 |
| 20 | 25 | 1 (0.2%) | 0 |
| Average ALS: 8.76 Average Controls: 8.19 | Average ALS: 14 Average Controls: 11.45 |  |  |
| Median ALS: 8 Median Controls: 8 | Median ALS: 11.5 Median Controls 10 |  |  |
